# Historical baselines of coral cover on tropical reefs as estimated by expert opinion

**DOI:** 10.7717/peerj.4308

**Published:** 2018-01-24

**Authors:** Tyler D. Eddy, William W.L. Cheung, John F. Bruno

**Affiliations:** 1 Nippon Foundation—Nereus Program, Institute for the Oceans & Fisheries, University of British Columbia, Vancouver, British Columbia, Canada; 2 Department of Biological Sciences, University of North Carolina at Chapel Hill, Chapel Hill, NC, United States of America

**Keywords:** Historical ecology, Marine environmental history, Qualitative survey, Expert opinion survey, Historical baselines, Shifting baseline syndrome, Climate change

## Abstract

Coral reefs are important habitats that represent global marine biodiversity hotspots and provide important benefits to people in many tropical regions. However, coral reefs are becoming increasingly threatened by climate change, overfishing, habitat destruction, and pollution. Historical baselines of coral cover are important to understand how much coral cover has been lost, e.g., to avoid the ‘shifting baseline syndrome’. There are few quantitative observations of coral reef cover prior to the industrial revolution, and therefore baselines of coral reef cover are difficult to estimate. Here, we use expert and ocean-user opinion surveys to estimate baselines of global coral reef cover. The overall mean estimated baseline coral cover was 59% (±19% standard deviation), compared to an average of 58% (±18% standard deviation) estimated by professional scientists. We did not find evidence of the shifting baseline syndrome, whereby respondents who first observed coral reefs more recently report lower estimates of baseline coral cover. These estimates of historical coral reef baseline cover are important for scientists, policy makers, and managers to understand the extent to which coral reefs have become depleted and to set appropriate recovery targets.

## Introduction

Since the publication of the seminal article, ‘Anecdotes and the shifting baseline syndrome’ (Pauly, 1995), it has been recognized that it is important to understand what was pristine prior to large, human-induced changes to the natural environment (Jackson et al., 2001; Lotze et al., 2006). Historical baselines are important for scientists, managers, policy makers, and the general public to understand how much a population or habitat has been impacted by humans through time (Johannes, 1981; Rosenberg et al., 2005; Eddy, Gardner & Pérez-Matus, 2010; McClenachan, Ferretti & Baum, 2012; Thurstan et al., 2015). Baselines can also provide realistic and meaningful targets for restoration (Pitcher, 2005). Yet determining a natural baseline is often difficult, in part because there is little data on which to base estimates, and also because different approaches can produce very different estimates (Bruno et al., 2014). Without them, the perception of what was pristine or natural has been shown to change with younger generations, as the reference of their earliest memories is different than that of their parents or grandparents (Sáenz-Arroyo et al., 2005; McClenachan, 2009).

Like many marine habitats, coral reefs have been drastically altered by human activities such as habitat destruction, nutrient and/or sediment runoff, overfishing, and increased frequency of bleaching events due to climate change. These impacts have resulted in significant declines in coral cover around the world (Bruno et al., 2009), including in the Caribbean Sea (Gardner & Co, 2003; Schutte, Selig & Bruno, 2010), and the Pacific Ocean (Gomez, Alcala & San Diego, 1981; Bellwood et al., 2004; Bruno & Selig, 2007). While it has been suggested that the number of people living within proximity of coral reefs explains the degree of coral degradation (Smith et al., 2016), recent research suggests that global scale actions are required to prevent further loss (Bruno & Valdivia, 2016). The loss of coral reefs has implications for tropical and subtropical coastal societies globally, as they provide habitat for species that are important for subsistence and indigenous fisheries (Cisneros-Montemayor et al., 2016).

Coral cover, the percentage of the seafloor occupied by living scleractinian corals, is an important metric of reef state or “health”. Coral cover is related to habitat volume and complexity and is a good predictor of fish and invertebrate diversity and abundance (Jones et al., 2004; Idjadi & Edmunds, 2006; Alvarez-Filip et al., 2009; Dustan, Doherty & Pardede, 2013). Coral cover also influences reef accretion; accretion rates generally decline with coral cover and on low cover reefs a greater portion of the carbonate reef structure is exposed to bioerosion (e.g., via urchin and parrotfish grazing) accelerating the loss of the reef framework (Perry et al., 2013; Kuffner & Toth, 2016).

Syntheses of quantitative benthic reef surveys (i.e., based on *in situ* assessments, video or still images) suggest coral cover began to decline in most regions in the late 1970s—early 1980s (Gardner & Co, 2003; Bruno & Selig, 2007; Schutte, Selig & Bruno, 2010). Unfortunately, using published estimations of coral coverage to extrapolate global trends may suffer from reporting biases. Particularly, reef monitoring only became common after noticeable coral decline had already begun: even at regional scales, the number of sites surveyed annually was low (<10) until relatively recently, e.g., until the mid 1980s for the Great Barrier Reef and the late 1990s for the Greater Caribbean (De’ath et al., 2012; Jackson et al., 2014; Schutte, Selig & Bruno, 2010; Gardner & Co, 2003; Bellwood et al., 2004). Although there are early quantitative estimates of coral cover from the late 1960s and early 1970s, only a very small number of quantitative surveys were performed globally in any given year (De’ath et al., 2012; Bruno & Selig, 2007; Jackson et al., 2014; Schutte, Selig & Bruno, 2010; Gardner & Co, 2003; Bellwood et al., 2004). Additionally, bias in site selection during this period could have influenced study outcomes. For example, scientists could have chosen reefs with extraordinary cover (or other characteristics) for aesthetic reasons. On the other hand, disturbance ecology was a popular research theme in the 1970s and the purpose of many of the quantitative surveys from the era was to document coral loss or low cover due to storms and predator outbreaks (and thus may have been biased against surveying high cover reefs). Overall, the inferences from long-term monitoring studies about coral cover and other characteristics of reefs in the absence of anthropogenic disturbances are unfortunately limited.

In the absence of systematic quantitative surveys predating human impacts on coral reefs, there have been a number of attempts to understand historical baselines of coral reefs. Although paleoecology has been applied for decades to measure changes in coral composition, e.g., in response to natural and anthropogenic disturbances (Aronson & Precht, 2001; Pandolfi et al., 2003; Wapnick, Precht & Aronson, 2004; Pandolfi & Jackson, 2006), this approach does not provide estimates of living coral cover. There have also been attempts to use photographic images to quantify coral reef percent cover prior to quantitative coral surveys (Clark et al., 2016); however, without a systematic survey, it is difficult to draw accurate conclusions about baselines of coral reef cover. An integrative approach combined archaeological deposits, ethnohistoric and anecdotal descriptions, and modern ecological and fishery data to evaluate the health of Hawaiian coral reefs over 700 years to document periods of reef decline and recovery (Kittinger et al., 2011). Interestingly, a recent study used navigational charts going back over 240 years to document a 52% decrease in patch reef cover in lagoons of the Florida Keys (McClenachan et al., 2017).

In the absence of quantitative data, drawing experience from coral reef experts through questionnaire surveys can help qualitatively estimate baselines. The “Reef Reminiscences” project gathered the memories of reef scientists of their work in the field half a century ago, painting a powerful picture of the state of reefs before most of their colleagues were born (Sale & Szmant, 2012). There have been approaches in the fisheries literature to reconstruct baselines of fish stocks using historical resources to document declines that were much greater than previously understood (Rosenberg et al., 2005). Additionally, fishers’ ecological knowledge has been used to estimate the baselines of various fish stocks and fisheries (e.g., Sadovy & Cheung, 2003; Teh et al., 2007; Eddy, Gardner & Pérez-Matus, 2010). Kleypas & Eakin (2007) surveyed reef scientists to measure their opinions about the relative importance of different threats to coral reef ecosystems. Also, expert opinion has been used to quantify the relative importance of different threats to different components of marine environment where information is limited, such as for the deep sea (MacDiarmid et al., 2012; Eddy, 2014), and to evaluate the effectiveness of fisheries management globally (Mora et al., 2009).

The purpose of this study was to estimate the coral cover baseline by assessing the opinions of coral reef scientists. We asked each participant what they believed “baseline coral cover” is. Our intent was that this value would be based on their own observations (which at the least would likely subconsciously bias their answer), their reading and interpretation of the literature, their discussions with colleagues, etc. We defined “baseline coral cover” as the global average pre-human impact, in shallow (1–15 m depth) fore reef environments. We also asked each participant questions related to their expertise and profession, level of experience, and the timing of their earliest field observations.

## Methods

To solicit as many as possible expert and ocean-user opinions about baselines of coral reefs, we designed a short, online survey that included four questions (Table 1). The survey was distributed via Twitter and the NOAA coral-list and remained open for three months (March 2–May 5, 2017). In both avenues, coral reef scientists, students studying coral reefs, managers, policy makers or NGO employees working in areas with coral reefs, and recreational divers who had dove on coral reefs were asked to fill out a quick survey. Due to the online nature of the survey, respondents were biased to those having access to email or the internet and are participants of the NOAA email coral list or the coral reef Twitter community or heard about it via word of mouth from someone who is. The Office of Human Research Ethics at the University of North Carolina at Chapel Hill determined that this submission did not constitute human subjects research as defined under federal regulations (45 CFR 46.102 (d or f) and 21 CFR 56.102(c)(e)(l)) and did not require IRB approval as communicated to JFB via email on March 1st, 2017 (Study # 17-0479).

**Table 1:** Questions asked in online survey. See ‘Methods’ for further details that were provided to participants.

	Question
1	What is your position?
2	What was the first year that you observed a coral reef?
3	What is the highest percent cover of a coral reef that you have observed? Where and when was this?
4	What is your baseline estimate of coral reef cover?

The first question asked the respondent about their job type in order to differentiate professional scientists, managers, policy makers, and non-governmental organization (NGO) employees, students, and recreational divers. The second question asked what was the first year that the respondent observed a coral reef (to estimate the level of experience and the personal baseline reference). The third question asked what was the highest coral cover observed and where and when this occurred.

The fourth question asked for an estimation of baseline percent coral cover. In this question, we stated: *“What would the average coral cover be in the absence of any human impacts in shallow (1–15 m depth) fore reef environments?”* We specified that *this is not the same as the maximum achievable coverage; even before humans, reefs were disturbed by storms, disease, etc. and thus some proportion of reefs would have been in various stages of recovery and not at peak coral cover.* We indicated that *“this value surely varies among regions, habitats, depths, etc; but still, we’d like your help in developing a consensus about what the historical average was. And by “human impacts” we mean both local and global impacts including fishing, pollution, and ocean warming. Finally, we are assuming that you will integrate your own observations with your knowledge of the literature, discussions with colleagues, etc.”*

We then summarized the responses by type of participant and grouped the location of the highest coral cover observed by region using joy or ridge line plots in *R*. The *ggjoy* package version 0.3.0 was used to compute and draw a kernel density estimate (https://github.com/clauswilke/ggjoy). We also plotted estimates of historical baseline coral cover and highest coral cover observed as a function of time to test for evidence of shifting baselines. Trend lines were calculated using the loess non-parametric local regression smoothing method in *R*.

**Figure 1:**
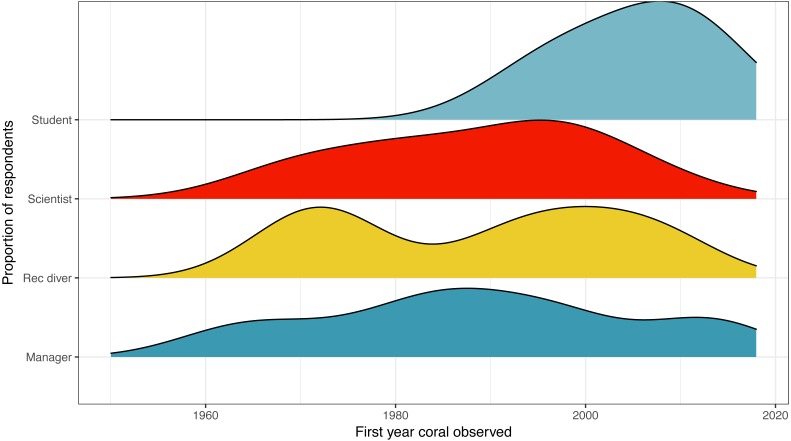
First year that a coral reef was observed by survey respondent position. Distribution of responses for first year that a coral reef was observed by survey respondent position. Colors are used to visually differentiate among positions. Numbers of respondents by position: professional scientists = 133, students = 45, managers, policy makers or NGO employees = 12, recreational divers (rec divers) = 5.

To situate our baseline coral cover estimate alongside coral cover reported in quantitative surveys, we extracted published coral cover survey data for the earliest years from each study and plotted alongside our baseline estimate. Survey data sources were: (De’ath et al., 2012; Bruno & Selig, 2007; Jackson et al., 2014; Schutte, Selig & Bruno, 2010; Gardner & Co, 2003; Bellwood et al., 2004). We have not plotted quantitative baselines for the other regions reported in our qualitative survey—Atlantic Ocean, Persian Gulf, and Red Sea; although there are some surveys for locations within each region (ex. Brazil in the Atlantic, Egypt in the Red Sea, and Bahrain in the Persian Gulf), it is unclear if these individual locations are representative of the wider region.

## Results

### Types of respondents

We received a total of 195 responses from 133 professional scientists (68%), 45 students (23%), 12 managers, policy makers or NGO employees (6%), and five recreational divers (3%).

### First year coral reef observed

The earliest year that a coral reef was observed was listed at 1960, the average year was 1992, while the latest year listed was 2016 (Fig. 1). For scientists, the earliest year was also listed as 1960, the average was 1988, and the latest year was 2013.

### Highest coral cover observed

**Figure 2:**
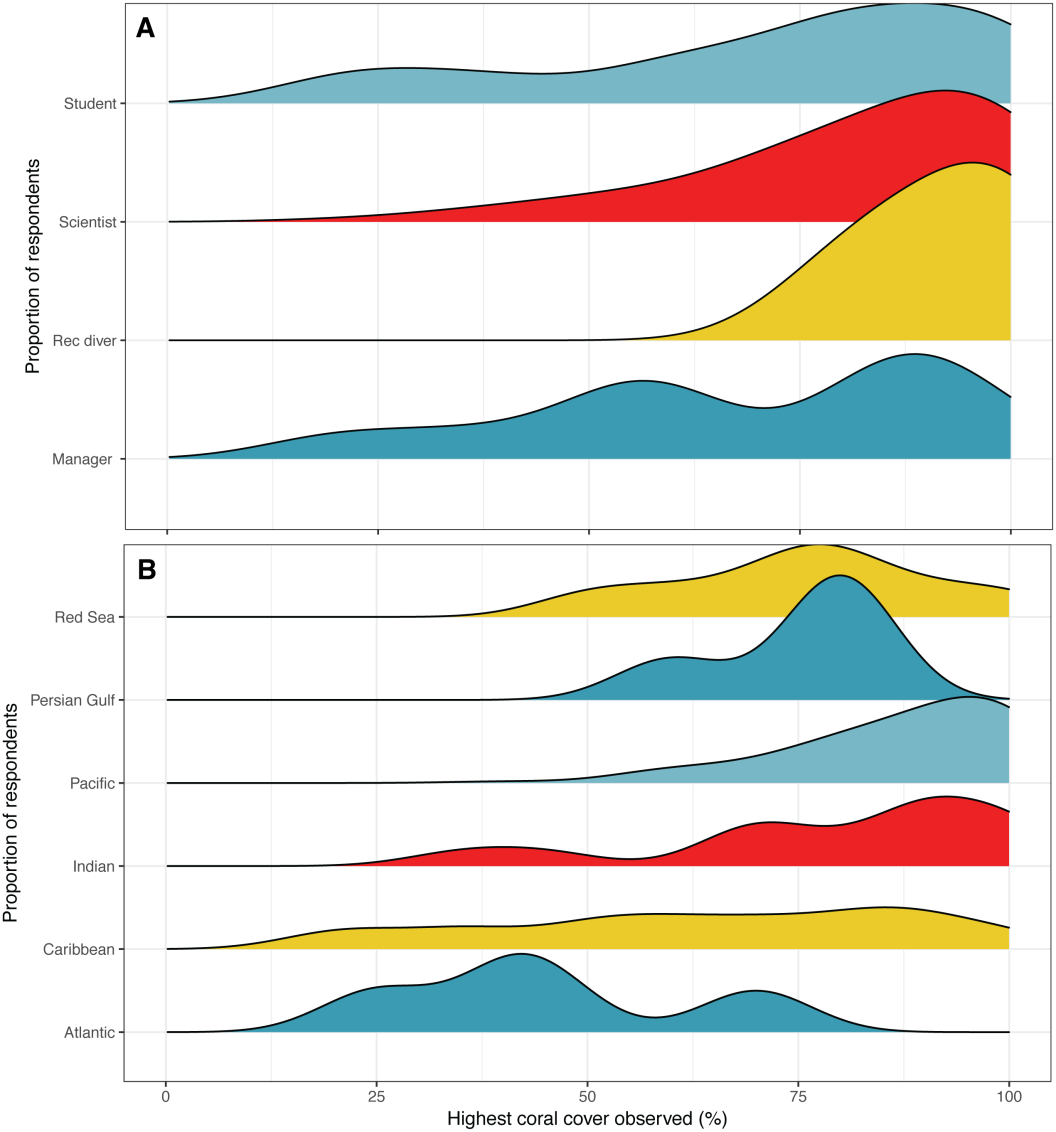
Highest coral cover observed. Distribution of responses for highest coral cover observed by (A) survey respondent position and (B) region. Colors are used to visually differentiate among positions. Numbers of respondents by position: professional scientists = 133, students = 45, managers, policy makers or NGO employees = 12, recreational divers (rec divers) = 5. Number of respondents by region: Atlantic Ocean = 5, Caribbean = 54, Indian Ocean = 13, Pacific Ocean = 109, Persian Gulf = 4, Red Sea = 8.

The average highest percent cover reported was 79% ± 21% SD (Fig. 2A). The earliest year that the highest percent cover observed by a respondent was 1962, the latest year reported was 2017. For scientists, the average highest coral percent cover was 82% ± 18% SD (Fig. 2A). The earliest year that the highest percent cover was observed was 1970, the latest year was also 2017, and the average highest percent cover observed in 2001.

Five respondents listed having observed the highest coral cover at locations in the Atlantic Ocean, 54 in the Caribbean, 13 in the Indian Ocean, 109 in the Pacific Ocean, four in the Persian Gulf, and eight in the Red Sea. Two respondents did not list a location. The highest average coral cover was observed at locations in the Pacific Ocean at 87%, followed by the Indian Ocean at 79%, 76% in the Red Sea, 75% in the Persian Gulf, 63% in the Caribbean Sea, and 45% in the Atlantic Ocean (Fig. 2B).

### Baseline estimates

**Figure 3:**
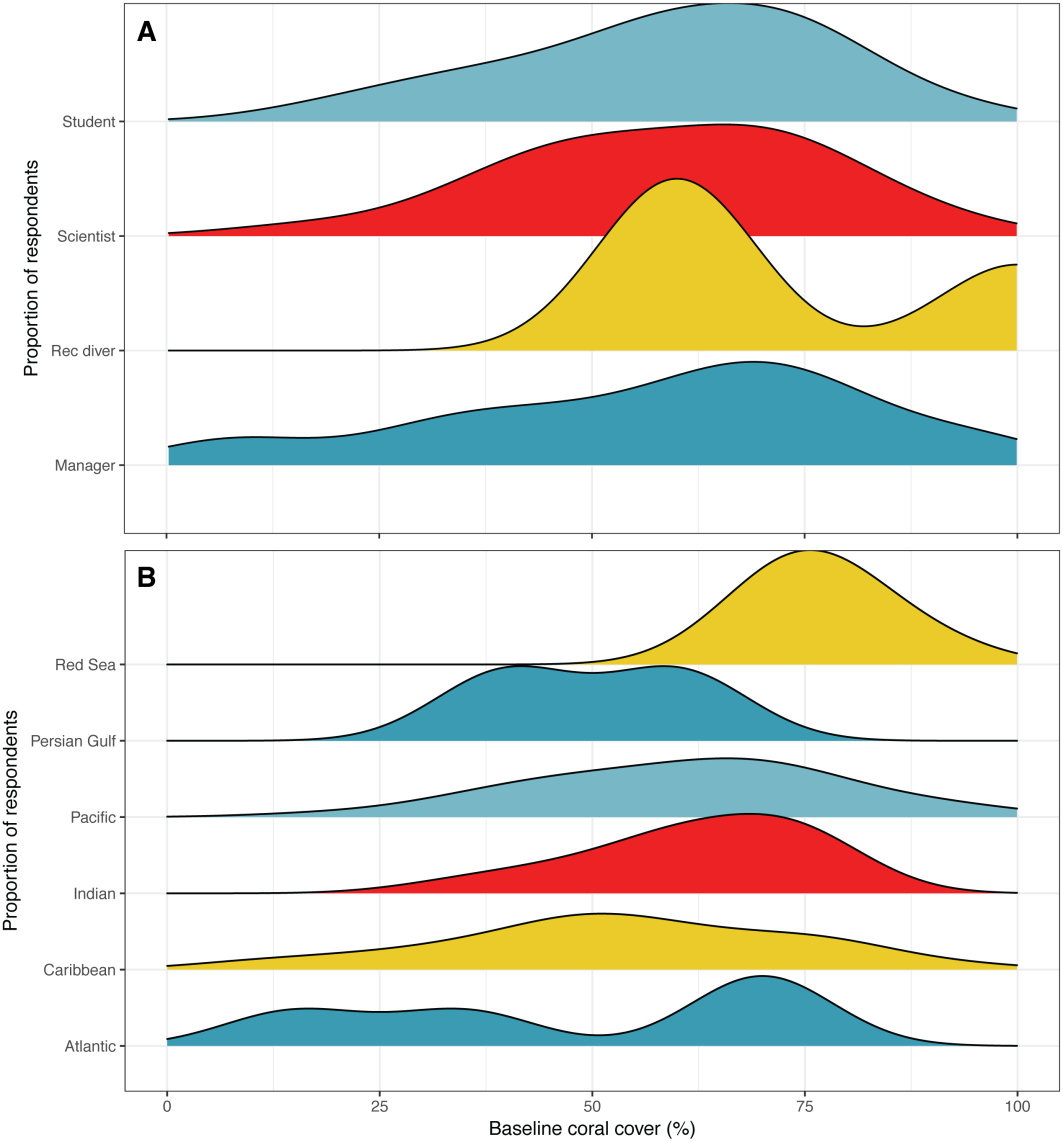
Estimates of baseline coral reef cover. Distribution of responses for expert opinion baseline estimates of coral reef cover by (A) survey respondent position and (B) according to the region where the respondent reported their highest observed coral cover. Colors are used to visually differentiate among positions. Numbers of respondents by position: professional scientists = 133, students = 45, managers, policy makers or NGO employees = 12, recreational divers (rec divers) = 5. Number of respondents by region: Atlantic Ocean = 5, Caribbean = 54, Indian Ocean = 13, Pacific Ocean = 109, Persian Gulf = 4, Red Sea = 8.

The mean estimate of baseline coral cover was 59% ± 19% SD, while for scientists it was 58% ± 18% SD (Fig. 3A). Interestingly, there were differences in baseline coral cover estimations according to the location that respondents had observed the highest coral cover. Respondents who indicated a location in the Red Sea as being the place where they had observed the highest percent cover estimated the highest baseline cover, with a mean of 77% (Fig. 3B). This was followed by an average estimate of 62% coral cover for the Indian Ocean, 61% for the Pacific Ocean, 53% for the Caribbean Sea, 50% for the Persian Gulf, and 48% for the Atlantic Ocean (Fig. 3B).

### Shifting baselines

**Figure 4:**
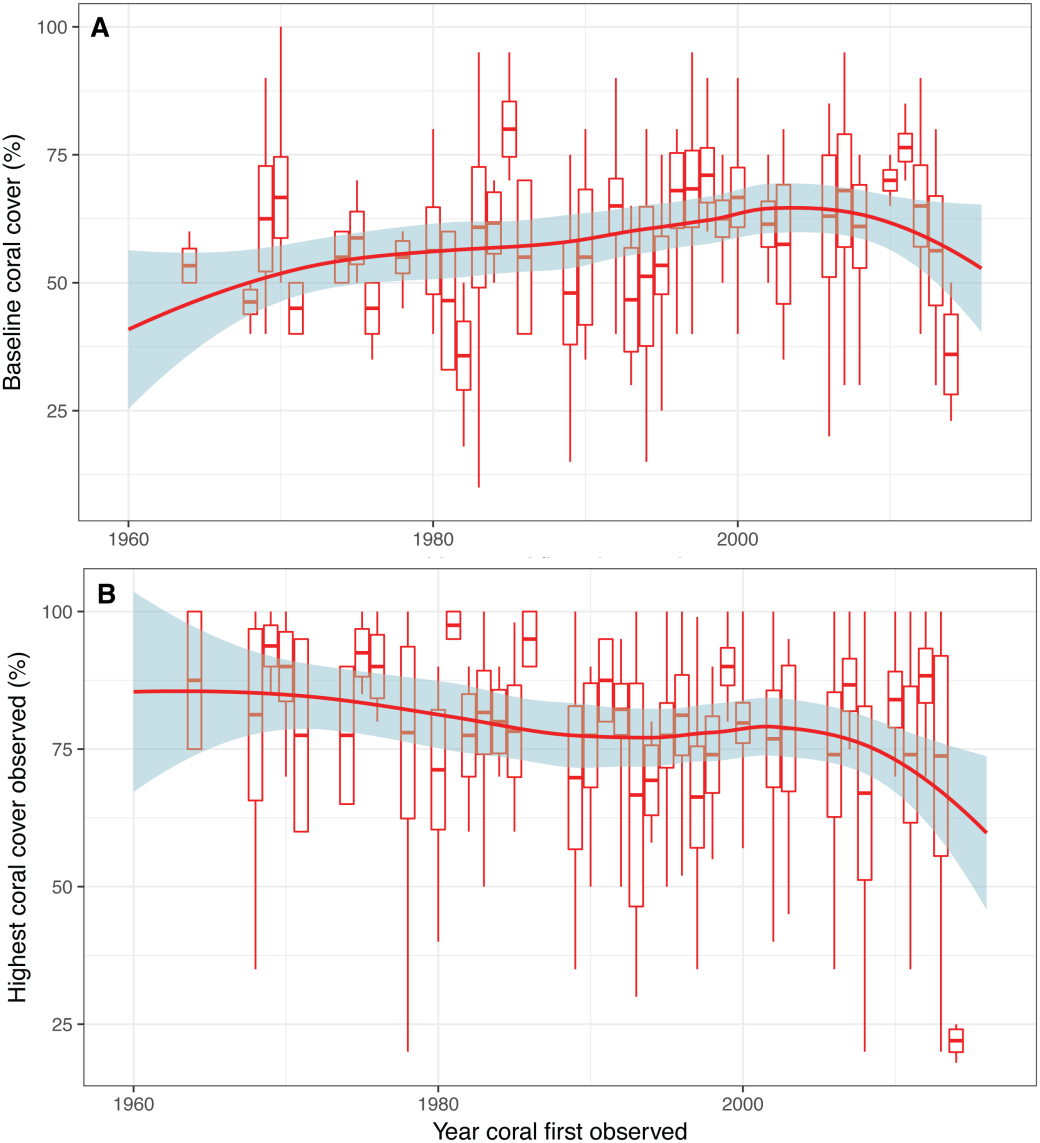
Baseline estimates and highest coral cover observed over time. (A) Expert opinion estimates of baseline coral cover and (B) highest coral cover observed as a function of the first year that a respondent observed a coral reef. Box plots depict mean with standard error and tails show the minimum and maximum values. Red trend line indicates smoothed conditional mean with confidence interval in blue.

We found no evidence of shifting baselines of coral reef cover as there is not a decline in the estimates of coral baselines with more recent first year of coral observations, and there appears to be a slight increase in baseline estimates over time (Fig. 4A). There was, however a decreasing trend in the highest coral cover observed as a function of time—which was represented by the first year of coral observation (Fig. 4B). These results indicate general decrease in highest observed coral coverage, but not the baseline.

### Qualitative vs. quantitative baselines

**Figure 5:**
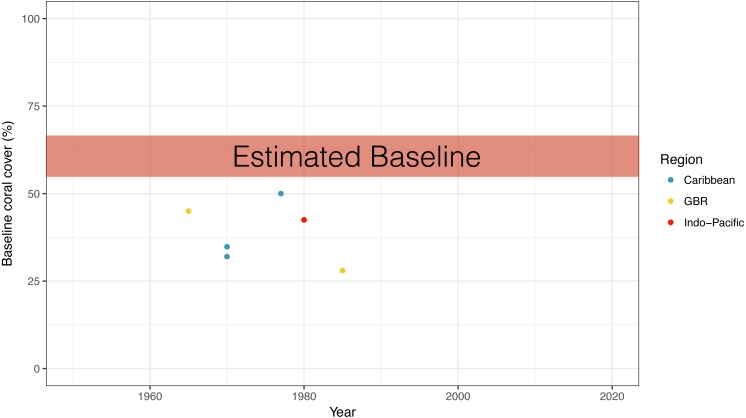
Qualitative vs. quantitative baselines. Comparison of the expert opinion estimated global coral cover baseline from this study to the earliest coral cover surveys in different ocean regions. References for quantitative studies are found in ‘Methods’. GBR, Great Barrier Reef.

Comparing the results from our baseline coral cover survey to published quantitative coral cover surveys dating back to the earliest years, we found that all quantitative surveys had lower values of coral cover (Fig. 5). The highest reported quantitative baseline value of 50% coral cover was reported from the Caribbean in 1977, while the lowest value was 28% from the Great Barrier reef in 1985 (Fig. 5).

## Discussion

Our expert opinion survey estimated global baseline coral cover at 59%, or 58% if only considering responses from non-student scientists. Baseline estimates varied depending on the region that respondents had observed the highest coral cover, such that estimates were the highest if a respondent had observed the highest coral cover in the Pacific, and lowest if observed in the Atlantic (Fig. 2B). These observations are in line with the ecology of the regions, as plating acroporid corals which can produce reefs with very high cover are not found in the Caribbean and some other regions. The global average baseline estimate from the survey appears to be biased by the proportion of respondents who reported to have observed corals in the Pacific who estimated a larger decline in coral cover relative to those reported to have observed corals in other regions. This is consistent with a previous survey that asked respondents to estimate baselines for the Indo-Pacific and the Caribbean in 2013, that also reported differences between the regions (Bruno, 2013). For the Caribbean, 40.4% of respondents estimated baseline coral percent cover between 25–50%, 38.4% of respondents estimated 50–75% cover, 17.7% estimated <25% cover, and 4.9% estimated >75% cover (Bruno, 2013). For the Indo-Pacific, 43.5% of respondents estimated coral cover baseline between 50–75%, 29% estimated >75% cover, 26.1% estimated between 25–50%, while 3.4% estimated <25% cover (Bruno, 2013).

An additional source of variation among regions are the natural disturbances that can affect coral cover such as predator outbreaks and storms, hurricanes, and bleaching events. The presence and relative frequency and magnitude of these and other natural disturbances varies among reefs and regions. For example, cyclonic storms are absent within ∼5% latitude of the equator and crown-of-thorns starfish (*Acanthaster planci*) are absent from the Caribbean. The degradation of some reefs appears to have begun centuries ago and it is very possible that human activities were already measurably affecting coral cover prior to the earliest field observations of our most experienced colleagues (Wing & Wing, 2001; Pandolfi et al., 2003), therefore we could still be underestimating the baseline.

The mean expert opinion baseline estimate is lower than the average highest percent cover reported and also lower than some surveys from the 1970s (e.g., Hughes, 1994; Sheppard et al., 2017). However, this seems reasonable because coral cover is reduced by natural disturbances including predators, storms, and diseases. Therefore, the baseline mean of a seascape or region would be substantially lower than the highest observed value, at least when integrated over time. Overestimating the cumulative impact (across larger scales of space and time than localized effects) of natural disturbances on coral cover could lead to an underestimate of the true baseline. On the other hand, it is possible that in the early years of reef science coral cover was atypically high, which could lead to an overestimate of the baseline. For example, Woodley (1992) argued that the high cover thickets of *Acropora* that dominated the reefs of Jamaica in the 1960s and 1970s were atypical and caused by an especially long lag in the return of large storms.

The lack of evidence for shifting baselines in survey respondents even with decrease in perceived coral coverage may indicate that spread of information/communication has generated consensus about historical baseline amongst the respondents. This may also suggest that the perceptions may be less independent among respondents. Either way, it appears that there is a general understanding among coral scientists and other surveyed groups that the baseline of coral reef percent cover was much greater than is generally reported today.

Expert opinion is a qualitative information source and will never replace quantitative data, however, in the absence of quantitative survey data dating to pre-industrial times, expert opinion is an invaluable resource. Recording the opinions of the relatively small number of scientists that worked on reefs when degradation was much less severe and widespread—from the 1950s to the early 1970s—should be considered a time sensitive priority (Sale & Szmant, 2012). There are of course, limitations to using expert opinion surveys, some of which we have addressed above related to geographical biases, as well as biases associated with recalled information from memory or personal experience which may be substantial (Daw, 2010). Additionally, we are unable to quantify the uncertainty associated with this historical baseline estimate of coral reef cover on tropical reefs. On the other hand, not only can expert opinion provide insight during time periods prior to quantitative data collection, they can also provide insight into parts of the ocean that have yet to be studied in depth, such as the deep sea (MacDiarmid et al., 2012; Eddy, 2014).

## Conclusions

Overall, we have provided an expert opinion estimate of the global coral percent cover baseline. While coral cover is presently declining at rapid rates around the world (Selig, Casey & Bruno, 2012; Bruno & Valdivia, 2016; Hughes et al., 2017a; Hughes et al., 2017b; Hughes et al., 2018), if we are capable of reversing these trends, it is important to understand what we should aim for as a target.

##  Supplemental Information

10.7717/peerj.4308/supp-1 Figure S1 Distribution of responses for first year that a coral reef was observed by survey respondent position Numbers of respondents by position: professional scientists = 133, students = 45, managers, policy makers or NGO employees = 12, recreational divers = 5.

10.7717/peerj.4308/supp-2 Figure S2 Distribution of responses for highest coral cover observed by survey respondent position Numbers of respondents by position: professional scientists = 133, students = 45, managers, policy makers or NGO employees = 12, recreational divers = 5. Number of respondents by region: Atlantic Ocean = 5, Caribbean = 54, Indian Ocean = 13, Pacific Ocean = 109, Persian Gulf = 4, Red Sea = 8.

10.7717/peerj.4308/supp-3 Figure S3 Distribution of responses for highest coral cover observed by region Numbers of respondents by position: professional scientists = 133, students = 45, managers, policy makers or NGO employees = 12, recreational divers = 5. Number of respondents by region: Atlantic Ocean = 5, Caribbean = 54, Indian Ocean = 13, Pacific Ocean = 109, Persian Gulf = 4, Red Sea = 8.

10.7717/peerj.4308/supp-4 Figure S4 Distribution of responses for expert opinion baseline estimates of coral reef cover by survey respondent position Numbers of respondents by position: professional scientists = 133, students = 45, managers, policy makers or NGO employees = 12, recreational divers = 5. Number of respondents by region: Atlantic Ocean = 5, Caribbean = 54, Indian Ocean = 13, Pacific Ocean = 109, Persian Gulf = 4, Red Sea = 8.

10.7717/peerj.4308/supp-5 Figure S5 Distribution of responses for expert opinion baseline estimates of coral reef cover according to the region where the respondent reported their highest observed coral cover Numbers of respondents by position: professional scientists = 133, students = 45, managers, policy makers or NGO employees = 12, recreational divers = 5. Number of respondents by region: Atlantic Ocean = 5, Caribbean = 54, Indian Ocean = 13, Pacific Ocean = 109, Persian Gulf = 4, Red Sea = 8.

10.7717/peerj.4308/supp-6 Supplemental Information 1 Qualitiative survey data Survey data provided by respondents about coral baselines, highest coral cover observed, location, position, and year coral first observed.

## Additional Information and Declarations

### Data Availability

The following information was supplied regarding data availability: The raw survey data has been provided as a Supplemental File.
