# Supplementary figures and images for: Historical baselines of coral cover on tropical reefs as estimated by expert opinion
Source: PeerJ. 2018 Jan 24;6:e4308. doi: 10.7717/peerj.4308 (PMC5786882; doi:10.7717/peerj.4308)

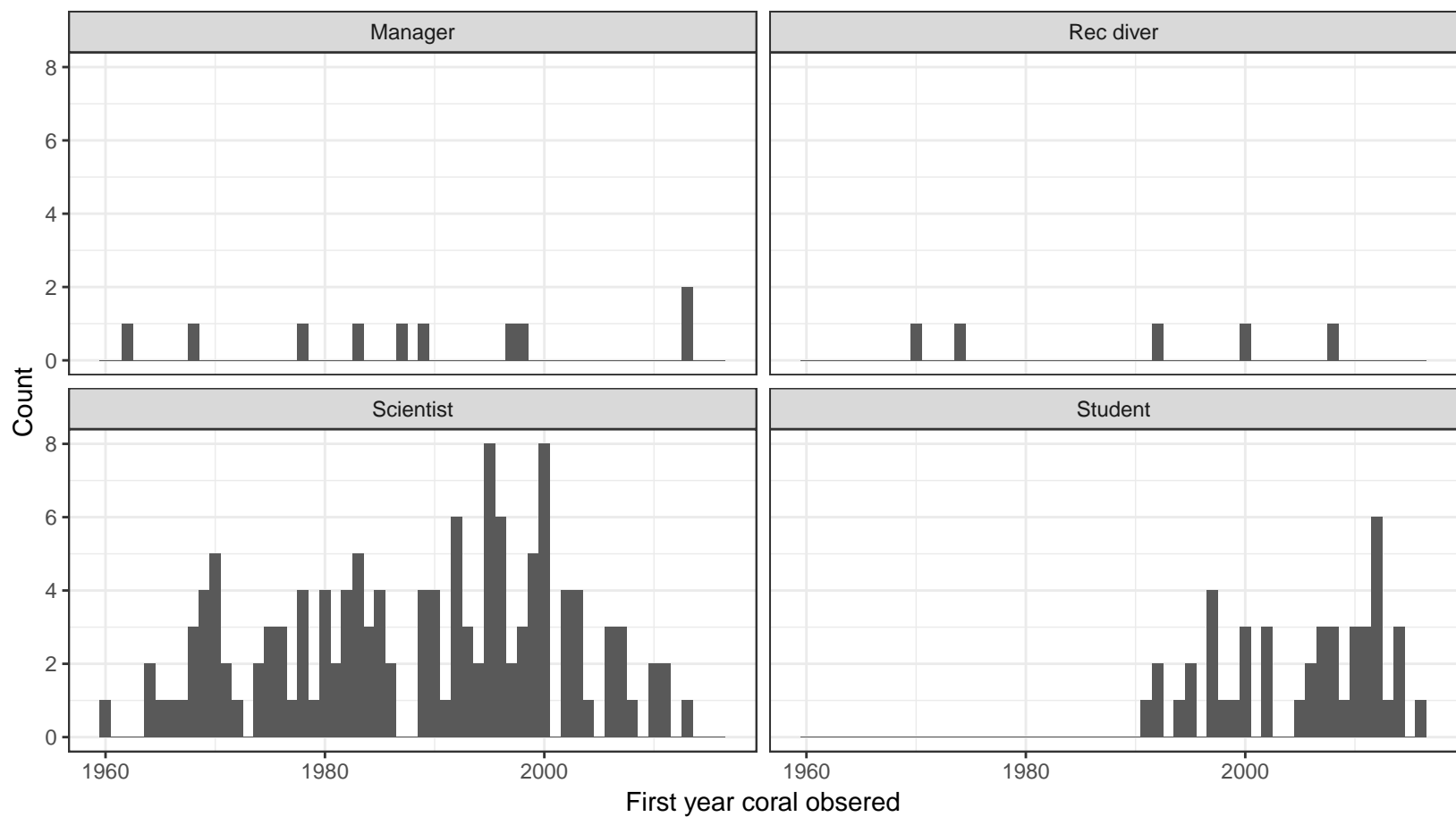

Supplement: Figure S1 — Numbers of respondents by position: professional scientists = 133, students = 45, managers, policy makers or NGO employees = 12, recreational divers = 5. [file peerj-06-4308-s001.pdf]

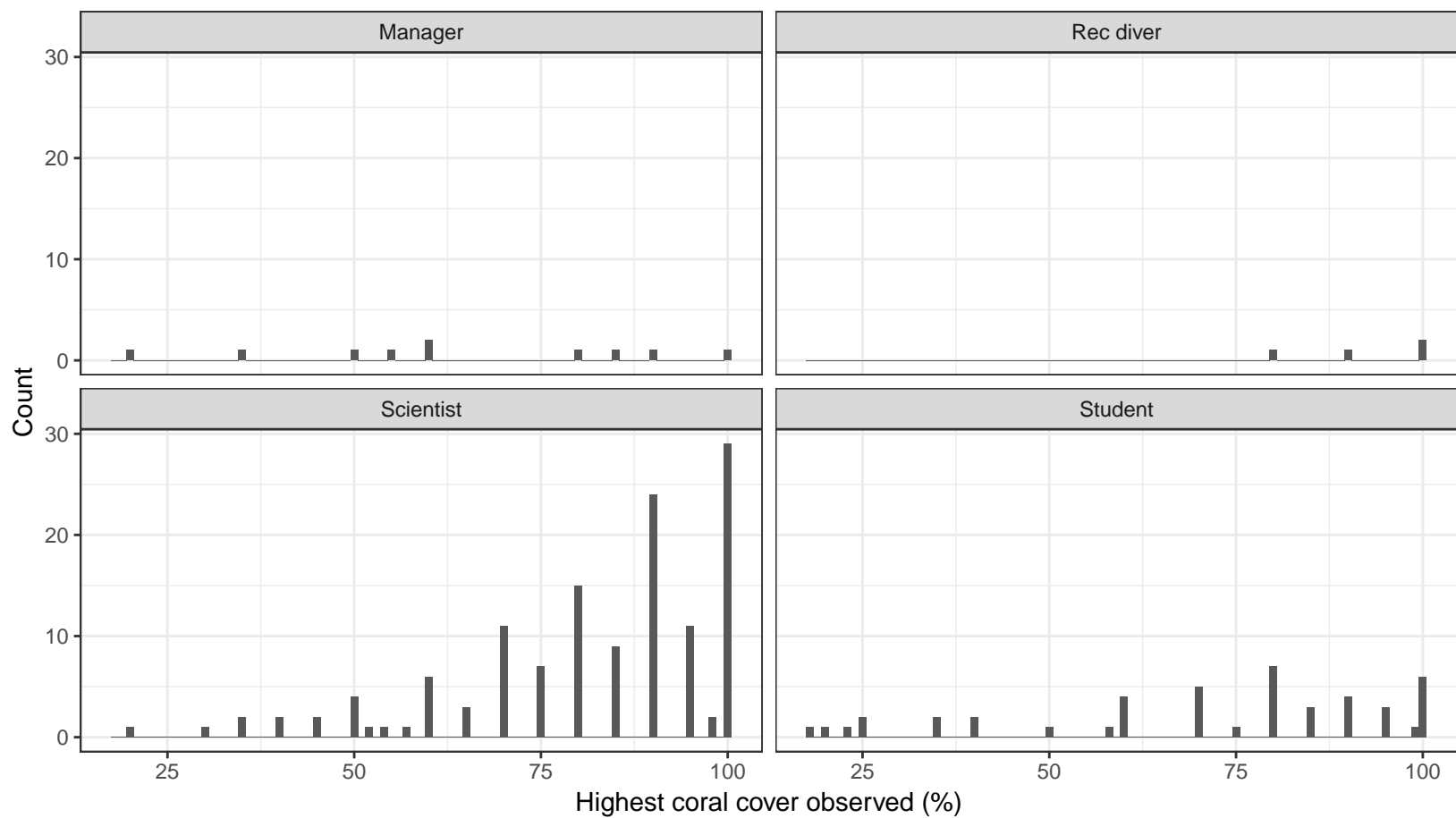

Supplement: Figure S2 — Numbers of respondents by position: professional scientists = 133, students = 45, managers, policy makers or NGO employees = 12, recreational divers = 5. Number of respondents by region: Atlantic Ocean = 5, Caribbean = 54, Indian Ocean = 13, Pacific Ocean = 109, Persian Gulf = 4, Red Sea = 8. [file peerj-06-4308-s002.pdf]

Count

Atlantic

30

20

10

0

Caribbean

Indian

Pacific

30

20

10

0

Persian Gulf

Red Sea

Highest coral cover observed (%)

25

50

75

100

25

50

75

100

25

50

75

100

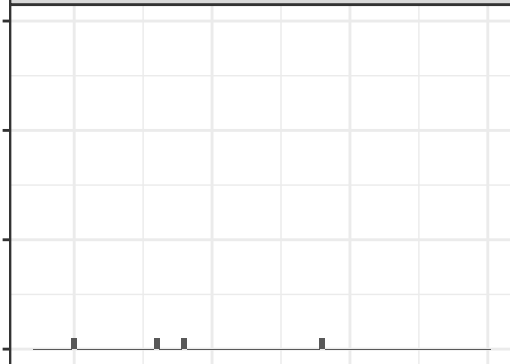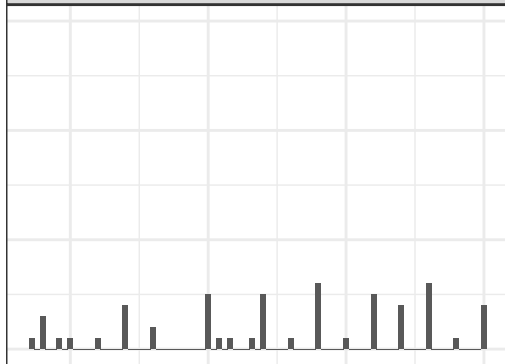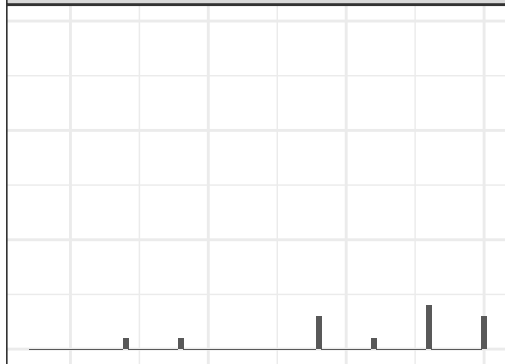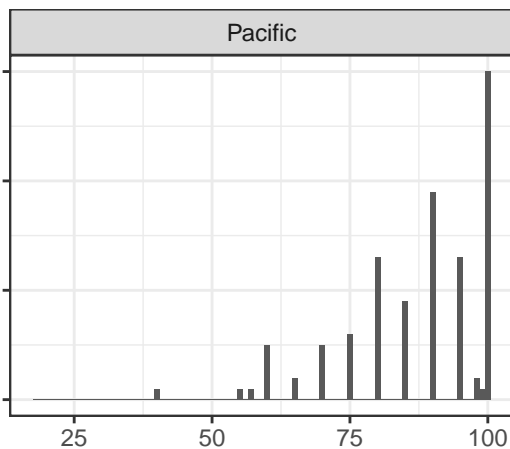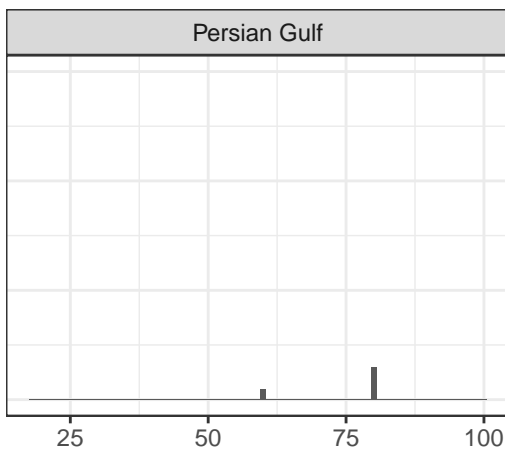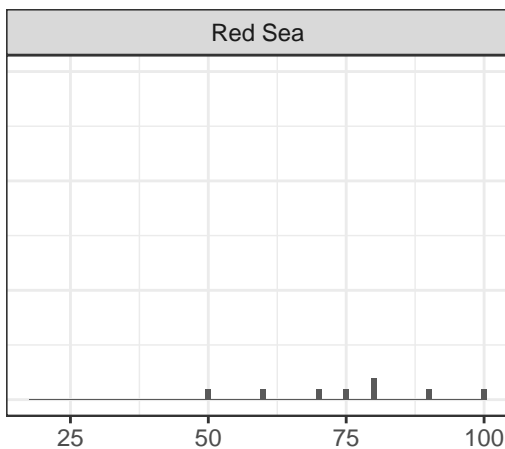

Supplement: Figure S3 — Numbers of respondents by position: professional scientists = 133, students = 45, managers, policy makers or NGO employees = 12, recreational divers = 5. Number of respondents by region: Atlantic Ocean = 5, Caribbean = 54, Indian Ocean = 13, Pacific Ocean = 109, Persian Gulf = 4, Red Sea = 8. [file peerj-06-4308-s003.pdf]

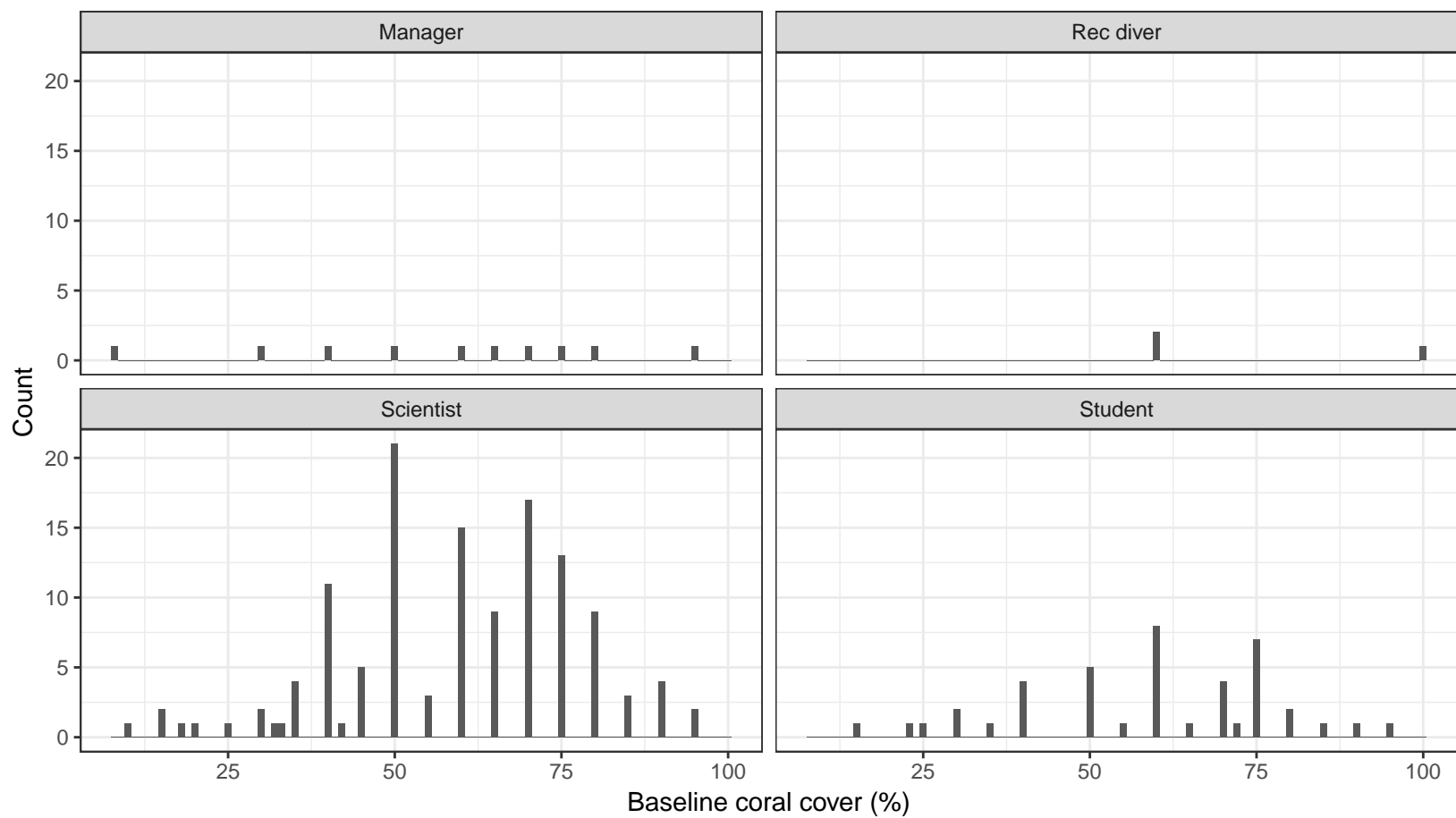

Supplement: Figure S4 — Numbers of respondents by position: professional scientists = 133, students = 45, managers, policy makers or NGO employees = 12, recreational divers = 5. Number of respondents by region: Atlantic Ocean = 5, Caribbean = 54, Indian Ocean = 13, Pacific Ocean = 109, Persian Gulf = 4, Red Sea = 8. [file peerj-06-4308-s004.pdf]

Count

Atlantic

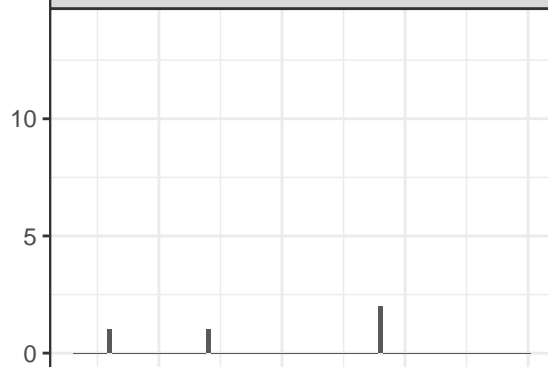

Caribbean

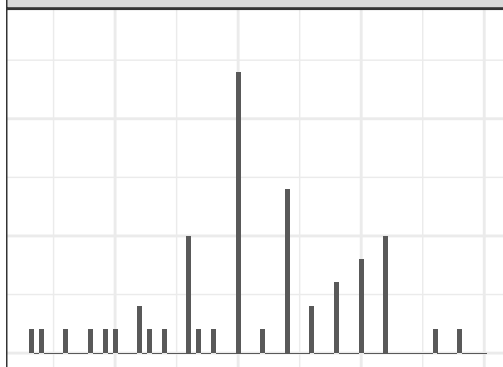

Indian

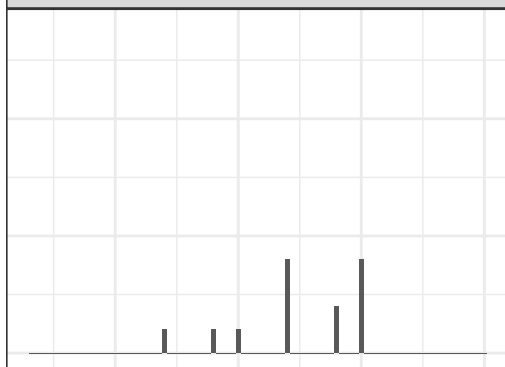

Pacific

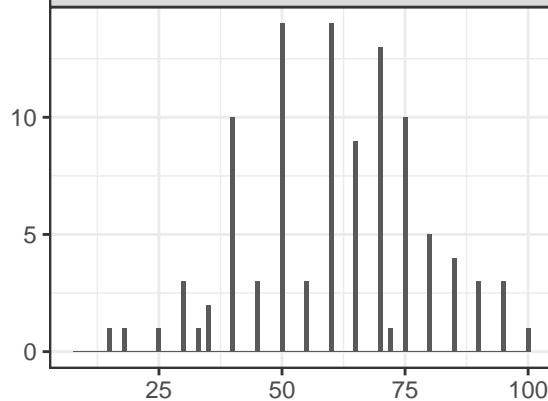

Persian Gulf

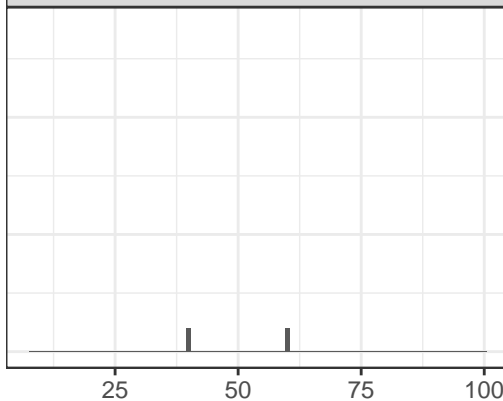

Red Sea

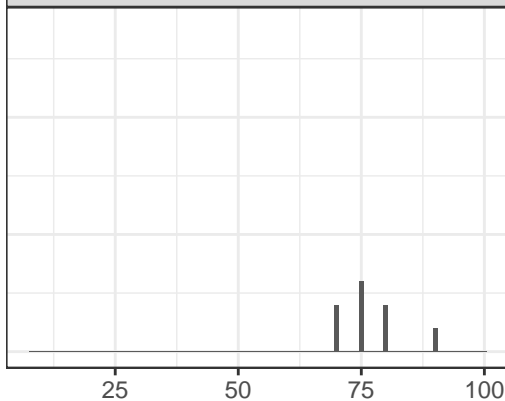

Baseline coral cover (%)

Supplement: Figure S5 — Numbers of respondents by position: professional scientists = 133, students = 45, managers, policy makers or NGO employees = 12, recreational divers = 5. Number of respondents by region: Atlantic Ocean = 5, Caribbean = 54, Indian Ocean = 13, Pacific Ocean = 109, Persian Gulf = 4, Red Sea = 8. [file peerj-06-4308-s005.pdf]
